# First Report of the Root-Knot Nematode, *Meloidogyne floridensis* Infecting Guardian® Peach Rootstock in South Carolina, USA

**DOI:** 10.21307/jofnem-2019-061

**Published:** 2019-09-17

**Authors:** Gregory L. Reighard, William G. Henderson, Sarah O. Scott, Sergei. A. Subbotin

**Affiliations:** 1 Clemson University, Department of Plant & Environmental Sciences, Clemson, SC, USA; 2 Clemson University Cooperative Extension Service, Edgefield, SC, USA; 3 Plant Pest Diagnostics Center, California Department of Food and Agriculture, Sacramento, CA, USA

**Keywords:** *Mesocriconema xenoplax*, *nad5* mtDNA gene, *Pratylenchus vulnus*, *Prunus persica*, *Xiphinema americanum*

## Abstract

In 2018 to 2019, soil and root samples from some declining peach orchards were collected in Edgefield County, South Carolina, USA. Excavated roots of Guardian® peach (*Prunus persica*) rootstock showed strong gall symptoms. Extracted root-knot nematodes (RKN) were identified by both morphological and molecular methods as *M. floridensis*. This is the first detection of the peach RKN in South Carolina and the third state in the USA after Florida and California.

The peach root-knot nematode (RKN), *Meloidogyne floridensis* (Handoo et al., 2004), is an important parasite that can severely impact commercial peach production because of its capability to overcome RKN resistance in peach rootstocks. This nematode species was first described in Florida in 2004 (Handoo et al., 2004) where it is currently found in 12 counties (Brito et al., 2015) and was recently detected in two counties in California (Westphal et al., 2019).

In 2018 to 2019, during surveys for nematodes in three declining peach orchards in Edgefield County, South Carolina, several plant parasitic nematodes including a RKN were found in soil and root samples. Excavated roots of *Prunus persica* showed strong gall symptoms (Fig. 1). Nematode species identification was performed using both morphological and molecular methods at the Plant Pest Diagnostics Center, California Department of Food and Agriculture, Sacramento, California. The RKN was identified as *Meloidogyne floridensis* in samples from two of the peach orchards. The rootstock used in these orchards was Guardian® peach, which is reportedly resistant to *M. incognita* and *M. javanica* (Nyczepir et al., 1999) but not to a Florida *Meloidogyne* isolate (Nyczepir and Beckman 2000), which later was described and named as *M. floridensis* (Handoo et al., 2004).

**Figure 1:**
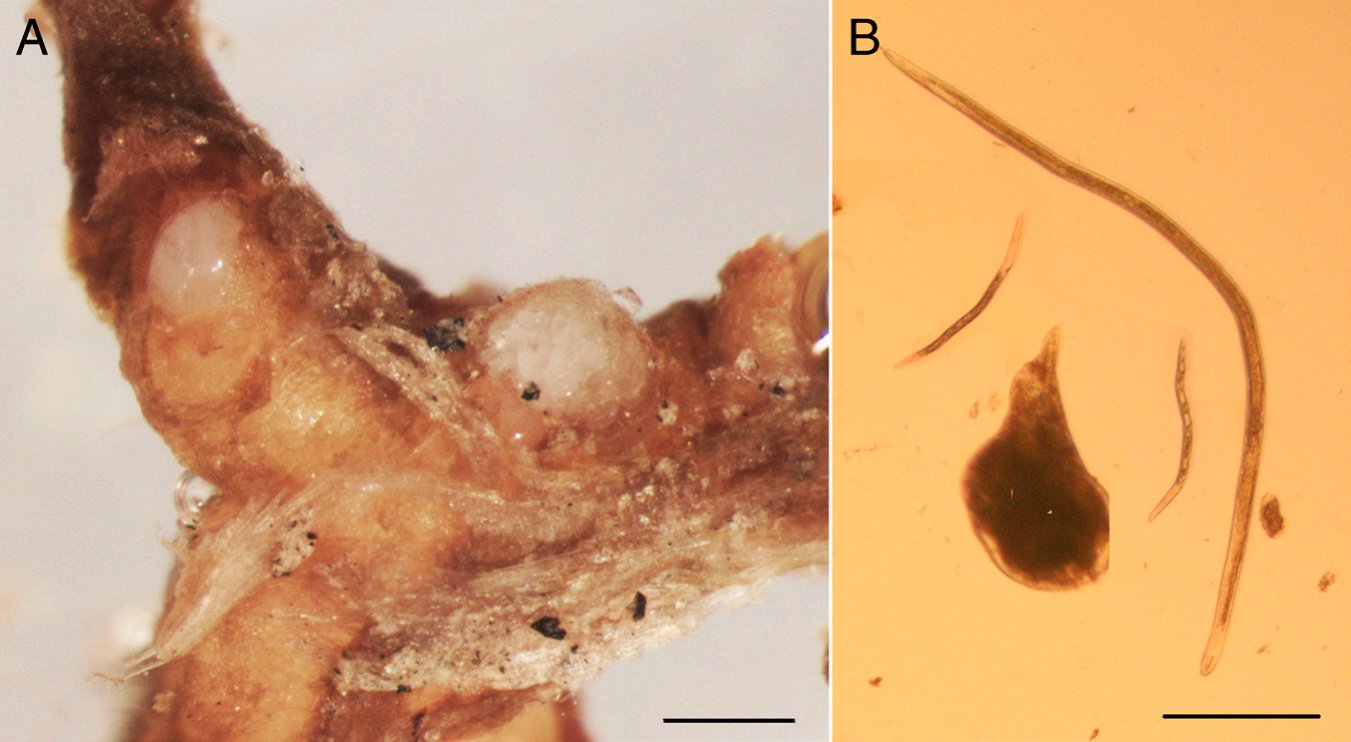
*Meloidogyne floridensis.* (A) Females on the Guardian® peach rootstock root; (B) Female, male and J2s and egg. Scale = 400 μm for A, 300 μm for B.

For light microscopy, several micrographs of different life stages of RNK were taken with an automatic Infinity 2 camera attached to a compound Olympus BX51 microscope equipped with Nomarski differential contrast (Fig. 2). Morphometric mean, standard deviation and range values of second-stage juveniles (J2s) of *M. floridensis* were (*n* = 5): *L* = 382 + 18.8 (363–405) μm; *W* = 16.0 + 0.6 (15.0–16.3) μm; *a* = 239 + 1.2 (22.3–24.9); *b* = 3.4 + 0.3 (2.8–3.8); *c* = 8.5 + 0.5 (8.0–9.2.9); stylet length = 12.5 + 0.4 (11.8–13.1) μm; center of median bulb to anterior end = 57.3 + 5.1 (52.5–65.0) μm; excretory pore to anterior end = 80.0 + 2.3 (77.5-82.5) μm; hyaline part of tail length = 11 + 1.0 (10.0–12.5) μm and tail length = 44.8 + 1.8 (42.5–47.5) μm. J2s had a smooth, truncated head and a tail tapering to a bluntly rounded terminus. Configuration of perineal patterns of females, morphology of males, morphology, and morphometrics of J2s of *M. floridensis* were mainly similar with those previously reported for isolates of this species from Florida (Handoo et al., 2004; Stanley et al., 2009) and California (Westphal et al., 2019). However, stylet of J2 from South Carolina was notably shorter than those of the Californian population and was similar with those of Florida population.

**Figure 2:**
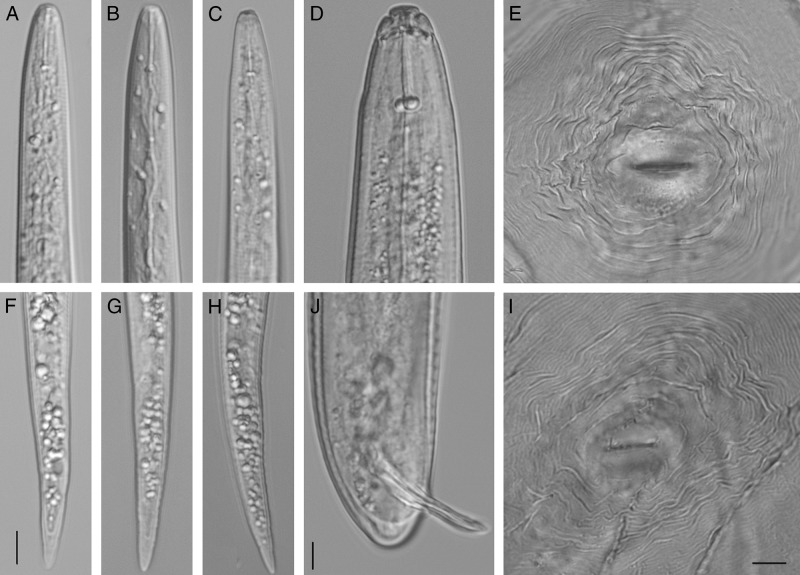
*Meloidogyne floridensis.* (A–C) Anterior region of J2s; (D) Head region of male; (F–H) Posterior region of J2s; (J) Posterior region of male; (E, I) Perineal patterns of females. Scale = 5 μm for A–D and F–J, 20 μm for E and I.

For molecular study, DNA was extracted from each stage of RKN: 10 J2, males and females, separately, using the proteinase K protocol. More than ten DNA samples were prepared and analyzed. Two primer sets were used: forward NAD5F2 (5′-TAT TTT TTG TTT GAG ATA TAT TAG-3′) and reverse NAD5R1 (5′-CGT GAA TCT TGA TTT TCC ATT TTT-3′) for the amplification of partial *nad*5 gene (Janssen et al., 2016) and forward C2F3 (5′-GGT CAA TGT TCA GAA ATT TGT GG-3′) (Powers and Harris, 1993) and M-flor-R2 (5′-ACA ATT GTT AAT TTA AAC AAC-3′) (original primer) for specific amplification of a short fragment of the mtDNA region between *COII* and 16S rRNA of *M. floridensis*. PCR products of these gene fragments were obtained and sequenced at Quintara Biosciences (San Francisco, CA). Sequences of *nad*5 gene (530 bp) and a short fragment between *COII* and 16S rRNA gene (99 bp) were identical to the reference and other sequences of these genes published for *M. floridensis* (Smith et al., 2015; Janssen et al., 2016; Westphal et al., 2019). Sequence of partial *nad5* gene was deposited in the GenBank under accession number MN072363. Thus, morphological and molecular results confirmed that the RKN infecting the Guardian® peach rootstock belongs to *M. floridensis.* To the best of our knowledge this is the first detection of the peach RKN in South Carolina, and the third state in the USA, after Florida and California. Several other plant-parasitic nematodes were identified using molecular methods in soil samples from three peach orchards: *Pratylenchus vulnus* (MN056433), *Xiphinema americanum* (MN072361, MN072362), *Paratrichodorus porosus,* (MN056434), *Mesocriconema xenoplax* (MN056431, MN056435), and *Tylenchorhynchus* sp. (MN056432).

## References

[ref001] Brito J. A., Dickson D. W., Kaur R., Vau S. and Stanley J. D. 2015 The peach root-knot nematode: *Meloidogyne floridensis*, and its potential impact for the peach industry in Florida. Nematology Circular 224:7.

[ref002] Handoo Z. A., Nyczepir A. P., Esmenjaud D., van der Beek J. G., Castagnone-Sereno P., Carta L. K., Skantar A. M. and Higgins J. A. 2004 Morphological, molecular, and differential-host characterization of *Meloidogyne floridensis* n. sp. (Nematoda: Meloidogynidae), a root-knot nematode parasitizing peach in Florida. Journal of Nematology 36:20–35. 19262784 PMC2620741

[ref003] Janssen T., Karssen G., Verhaeven M., Coyne D. and Bert W. 2016 Mitochondrial coding genome analysis of tropical root-knot nematodes (*Meloidogyne*) supports haplotype based diagnostics and reveals evidence of recent reticulate evolution. Scientific Reports 6:22591. 26940543 10.1038/srep22591 PMC4778069

[ref004] Nyczepir A. P. and Beckman T. G. 2000 Host status of Guardian peach rootstock to *Meloidogyne* sp. and *M. javanica*. HortScience 35(4):772.

[ref005] Nyczepir A. P., Beckman T. G. and Reighard G. L. 1999 Reproduction and development of *Meloidogyne incognita* and *M. javanica* on Guardian peach rootstock. Journal of Nematology 31:334–40. 19270905 PMC2620375

[ref006] Powers T. O. and Harris T. S. 1993 A polymerase chain reaction method for identification of five major *Meloidogyne* species. Journal of Nematology 25:1–6. 19279734 PMC2619349

[ref007] Smith T., Brito J. A., Han Y., Kaur R., Cetintas R. and Dickson D. W. 2015 Identification of the peach root-knot nematode, *Meloidogyne floridensis*, using mtDNA PCR-RFLP. Nematropica 45:138–43.

[ref008] Stanley J. D., Brito J. A., Kokalis-Burelle N., Frank J. H. and Dickson D. W. 2009 Biological evaluation and comparison of four Florida isolates of *Meloidogyne floridensis*. Nematropica 39:255–71.

[ref009] Westphal A., Maung Z. T. Z, Doll D. A., Yaghmour M. A., Chitambar J. J. and Subbotin S. A. 2019 First report of the peach root-knot nematode, *Meloidogyne floridensis* infecting almond on root-knot nematode resistant ‘Hansen 536’ and ‘Bright’s Hybrid 5’ rootstocks in California, USA. Journal of Nematology 51(1–3): e2019-02, doi: 10.21307/jofnem-2019-002. PMC6929654 31115204

